# Fluid Therapy and the Microcirculation in Health and Critical Illness

**DOI:** 10.3389/fvets.2021.625708

**Published:** 2021-05-13

**Authors:** Edward S. Cooper, Deborah C. Silverstein

**Affiliations:** ^1^Department of Veterinary Clinical Sciences, Ohio State University College of Veterinary Medicine, Columbus, OH, United States; ^2^Department of Clinical Studies and Advanced Medicine, University of Pennsylvania School of Veterinary Medicine, Philadelphia, PA, United States

**Keywords:** microcirculation, macrocirculation, shock, sepsis, hemorrhage, glycocalyx

## Abstract

Fluid selection and administration during shock is typically guided by consideration of macrovascular abnormalities and resuscitative targets (perfusion parameters, heart rate, blood pressure, cardiac output). However, the microcirculatory unit (comprised of arterioles, true capillaries, and venules) is vital for the effective delivery of oxygen and nutrients to cells and removal of waste products from the tissue beds. Given that the microcirculation is subject to both systemic and local control, there is potential for functional changes and impacts on tissue perfusion that are not reflected by macrocirculatory parameters. This chapter will present an overview of the structure, function and regulation of the microcirculation and endothelial surface layer in health and shock states such as trauma, hemorrhage and sepsis. This will set the stage for consideration of how these microcirculatory characteristics, and the potential disconnect between micro- and macrovascular perfusion, may affect decisions related to acute fluid therapy (fluid type, amount, and rate) and monitoring of resuscitative efforts. Available evidence for the impact of various fluids and resuscitative strategies on the microcirculation will also be reviewed.

## Introduction

Given the complex and multi-faceted nature of emergent and critical disease processes, determination of optimal approaches to fluid resuscitation continues to be a challenge for both human and veterinary medicine. Further complicating the ideal approach is determining how best to initially assess the severity of systemic compromise and gauge response to therapy. Traditionally, monitoring of a patient's hemodynamic state has been conducted at the macrovascular level by monitoring heart rate, respiratory rate, blood pressure, oxygen content and lactate, among other parameters. While these parameters may reflect systemic cardiovascular regulation, they do not necessarily indicate what is occurring at the level of the microvasculature. As the microcirculation is ultimately the conduit for delivery of oxygen and nutrients to tissues, it represents a relatively uncharted avenue of exploration to help enhance our understanding of disease processes like trauma and sepsis, its impact on tissue perfusion, as well as new ways to monitor response to therapy.

The goal of this manuscript will be to explore the structure, function, and regulation of the microcirculation, both in health and in response to disease processes like traumatic hemorrhage (representing hypovolemia and systemic vasoconstriction) and sepsis (representing a vasodilatory and maldistributive process). Finally, there will be consideration of modalities for assessing the microcirculation and tissue perfusion.

## Structure and Function of the Microcirculation

**Figure 1:**
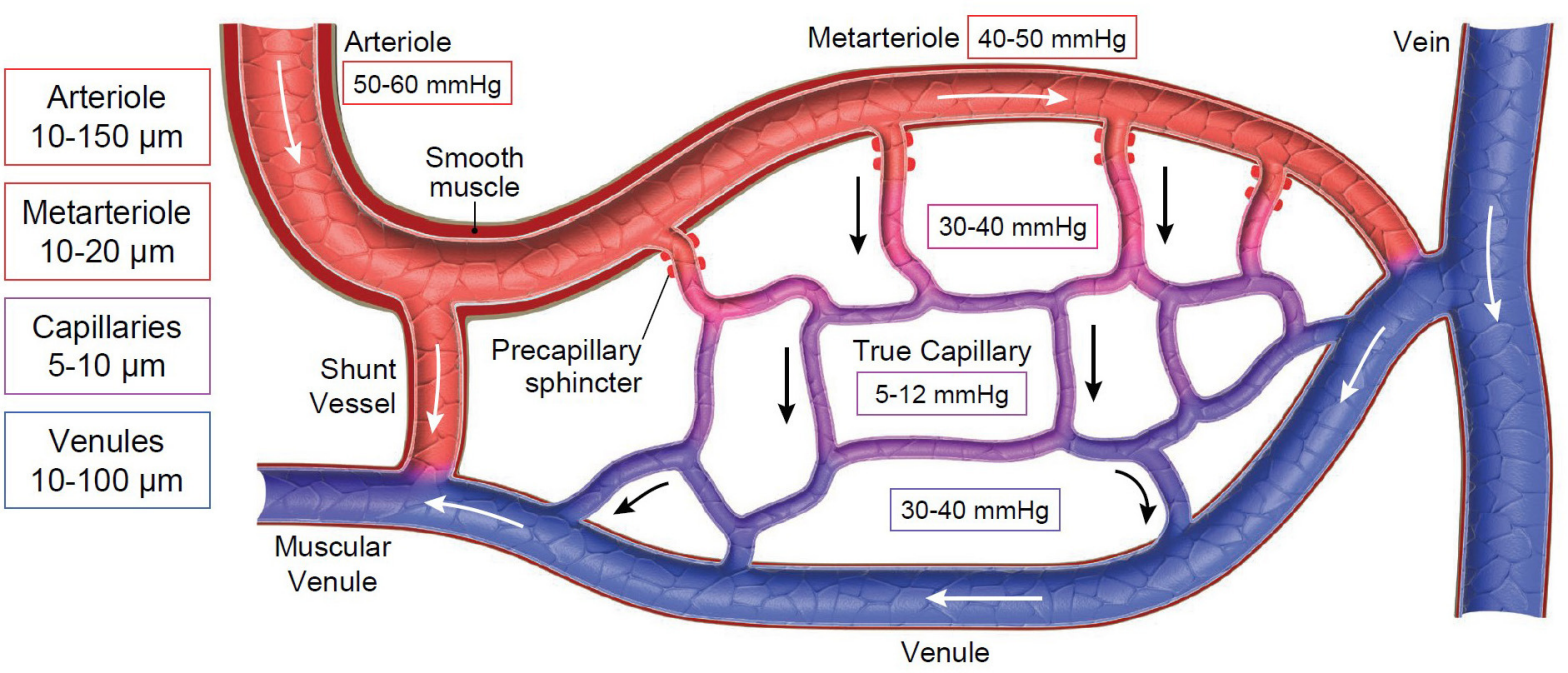
Schematic of the microcirculation. Boxes on the left represent ranges of vessel diameters at varying levels of the microcirculation. Boxes throughout the diagram represent the average interstitial (tissue) oxygen tension (P_t_O_2_). Arrows represent direction of blood flow across the microcirculatory unit.

The microcirculatory unit is comprised of arterioles feeding into a capillary bed that is drained by venules (Figure 1). The feeder arterioles are highly muscular throughout their length, while the terminal metarterioles have intermittent bands of smooth muscle. True capillaries have walls that lack musculature and are one endothelial cell thick and attached to a basement membrane. A precapillary sphincter is located between the arteriole and the capillary bed. Arterioles and venules in the microcirculation generally have a diameter <100 microns, while the capillaries are <10 microns in diameter (1). There are also shunt vessels that allow arterial blood to completely bypass the associated microcirculatory unit, as dictated by arteriolar and precapillary sphincter tone (1). The vascular endothelial surface layer (ESL) also plays a pivotal role in health and disease; this is the intimal surface of blood vessels containing the endothelial glycocalyx and associated components from the endothelial cells and plasma (2, 3). The ESL ranges from 200 nm to 2 μm in thickness and comprises up to 25% of the vascular space (4). The glycocalyx is a complex carbohydrate rich gel-like layer that serves as a barrier between the vessel wall and the blood (2, 5, 6) and has an overall negative charge. It contains membrane-bound proteoglycans, secreted glycosaminoglycans, sialic acid-containing glycoproteins, and glycolipids that are associated with the vascular endothelial surface (7). Proteins within the plasma, such as albumin and antithrombin, are also contained within the glycocalyx (7–9). The primary goals of the ESL are to maintain the vascular permeability barrier, modulate nitric oxide produced in response to shear stress, retain protective enzymes such as superoxide dismutase, contain factors that inhibit coagulation such as antithrombin, tissue factor pathway inhibitor and protein C, assist with mechanotransduction, and prevent leukocyte adhesion and binding of ligands to control local inflammation (3, 10). Recent discoveries in the ESL have contributed to the revised version of the Starling principle of transvascular fluid flux (11). Further details about the ESL are described in “Resuscitative Fluid Therapy and the Endothelial Surface Layer” found elsewhere in this “Fluid Therapy in Small Animals” series.

Altogether, the microcirculation represents the largest vascular surface area in the body and is vital for the effective delivery of nutrients to the cells and removal of waste products from the tissue beds (1). Both systemic and local regulation of blood flow through these units, as well as maintenance of the ESL, are essential to maintain adequate perfusion and match metabolic demand to oxygen/nutrient delivery.

## Microvascular Perfusion—Systemic Control

Vascular tone can be influenced by numerous chemical mediators in the circulation, such as catecholamines, endothelin and thromboxane (Table 1). Catecholamines are important (predominantly) vasoconstrictor agents released in large amounts when the sympathetic nervous system is activated. This occurs via central nervous system control in response to baroreceptors and chemoreceptors in the aortic arch and carotid sinuses (12). Baroreceptors respond to changes in vessel wall distension and chemoreceptors respond to hypoxia, hypercapnia or acidemia with associated modulation in vascular tone. These effects are most important once the systemic arterial pressure drops below 80 mmHg; they attempt to maintain perfusion in individual capillary beds despite decreased systemic blood pressure (12).

**Table 1:** Endogenous chemical mediators of vascular smooth muscle tone.

**Vasoconstriction**	**Vasodilation**
Thromboxane A2	Prostacyclin
Endothelins	Endothelium-derived hyperpolarizing factor
Endothelium-derived constricting factor 1	Nitric oxide
Endothelium-derived constricting factor 1	Histamine
Vasopressin	Kinins
Angiotensin II	Carbon dioxide
Epinephrine/Norepinephrine	Elevated tissue potassium, ADP, adenosine
Hypothermia	Hyperthermia
Hyperoxia	Hypoxia
Alkalosis	Acidosis

It is important to note sympathetic innervation exists for the arterial tree and much of the venous system but is not present down to the level of the capillaries. The absence of innervation and musculature in the true capillaries means flow through each capillary bed is regulated by the hemodynamic pressures generated between the precapillary sphincter and the post capillary venules. A single capillary bed can be fed by multiple arterioles, a situation that allows flow through the capillary bed to increase by 200–500% without any significant change in the overall arteriolar pressure (13). This can help to preserve microcirculatory flow to specific tissue beds during periods of transient systemic hypotension.

Maintenance of vascular tone is not the only factor influencing arterial blood pressure and tissue perfusion. Vascular volume also contributes to maintenance of appropriate blood pressure and tissue perfusion. In normal animals, the renin-angiotensin-aldosterone system is integral in maintaining appropriate blood volume, as well as contributing to systemic vascular tone (4). Angiotensin II acts on vascular smooth muscle to cause vasoconstriction and in the proximal tubules it causes sodium and water retention, leading to increased blood volume and arterial pressure (14). Angiotensin also causes the release of aldosterone and vasopressin, thus promoting vasoconstriction and water retention (14).

Systemically acting vasodilators include the kinins, adrenomedullin and atrial natriuretic peptide (ANP). The kinins include bradykinin and l-lysyl-bradykinin and are activated by the kallikreins. They typically regulate blood flow at the tissue level but can also be found circulating in the bloodstream. In addition to causing arteriolar dilation, bradykinin increases capillary permeability, thereby increasing the delivery of nutrients to the tissue bed (15). Histamine, which is released when tissues are damaged or in allergic reactions, also acts as a vasodilator and increases capillary permeability. Adrenomedullin increases the production of NO while ANP acts to antagonize numerous vasoconstrictor agents, both exerting their influence on the vascular tone indirectly (15).

These systemic regulators are responsible for controlling delivery of blood to the precapillary sphincter across different tissue beds. Once blood arrives at the capillary bed, local regulatory mechanisms act to maintain flow through the capillary bed, sometimes independent of systemic changes to perfusion (15).

## Microvascular Perfusion—Local Control

Basal tissue bed requirements vary based on their metabolic rate, nutrient availability and accumulation of waste products. Given that each capillary bed has unique requirements that may change independent of nearby capillary beds or systemic tissue needs, there are many local regulators of microcirculatory flow (Table 1). Typically, these changes in perfusion occur at the level of the precapillary sphincter. Independent regulation of flow based on local tissue needs results in selective capillary perfusion and the potential for microcirculatory shunting. Given the expansive nature of the capillary circulation, cardiac output would be insufficient to maintain forward flow if every capillary bed were to open simultaneously. Thus, the ability to adjust capillary perfusion through local and systemic changes is essential for modulating cardiac workload (15).

**Figure 2:**
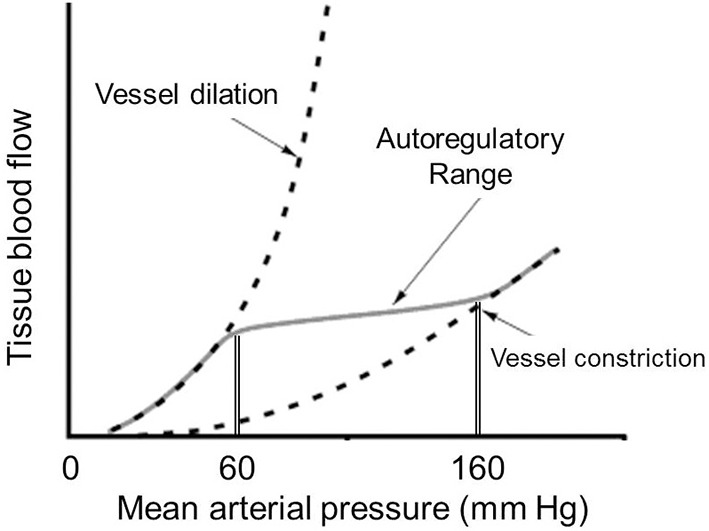
Curve reflecting tissue autoregulation for maintaining consistent blood flow across varying systemic perfusion pressures.

Rapid control of microcirculatory flow is mediated at a local level through autoregulation. One major mechanism is flow autoregulation, which allows maintenance of consistent capillary blood flow over a wide range of arterial perfusion pressure. This is achieved through vascular stretch receptors which respond to changes in peripheral pressure. Increases in vascular pressure trigger increased tone of the precapillary sphincter to mute transmission of that pressure though the capillary circuit (16). The opposite happens with a fall in peripheral pressure. This mechanism is effective for maintaining consistent flow over a perfusion pressure (mean arterial pressure) of 60–160 mm Hg and occurs independent of any neurohormonal input (Figure 2).

Changes in local metabolic demand can also have a significant impact on local control of blood flow. Understandably, by-products of increased metabolic activity, such as carbon dioxide, lactate, and hydrogen ion will trigger vasodilation to enhance local blood flow and improve oxygen/nutrient delivery. While these metabolites are produced downstream, the countercurrent flow between arterioles and venules allows them to be “sensed” at the level of the precapillary sphincter and alter inward flow (16). In addition, inter-cell and local neural pathways allow conduction of signals from capillary endothelium and venular smooth muscle in response to these signals (16).

**Figure 3:**
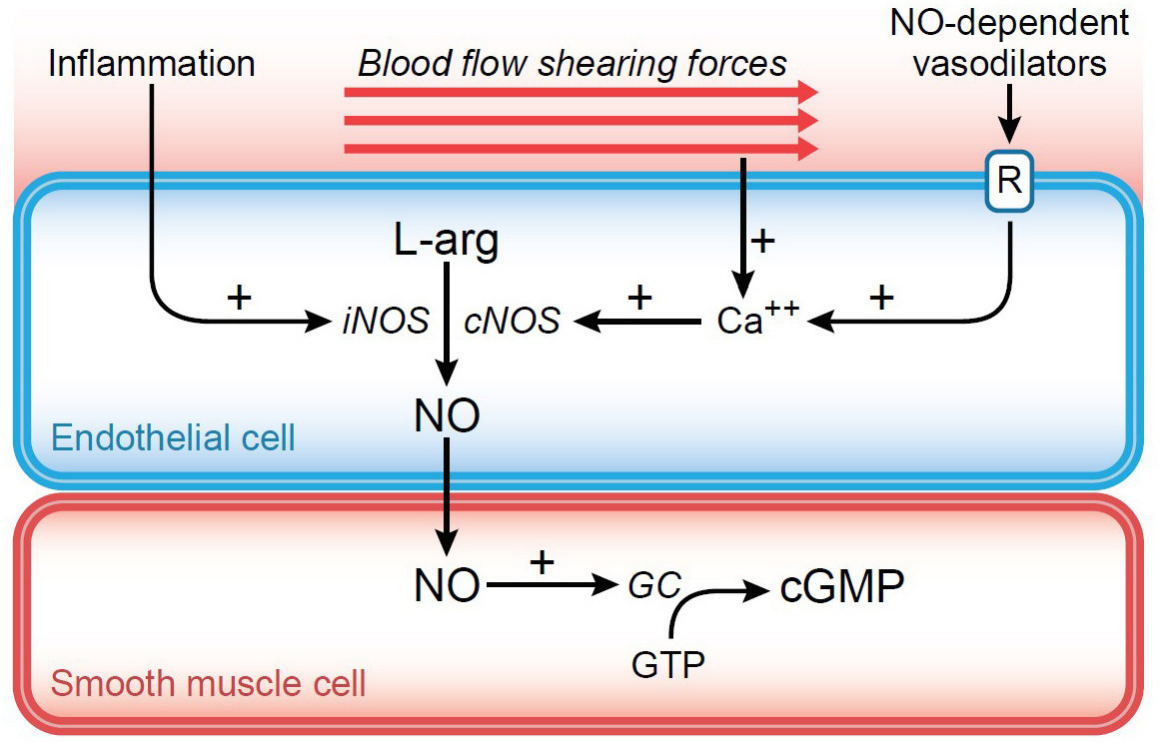
Diagram representing the impact of shear force generated against the endothelium. Through mechanotransduction, intracellular calcium is increased leading to increased nitric oxide (NO) production from stimulation of constitutive nitric oxide synthetase (cNOS). NO diffuses into surrounding smooth muscle cell causing activation of guanylyl cyclase (GC) and conversion of guanosine triphosphate (GTP) into cyclic guanosine monophosphate (cGMP). The resulting decrease in cytosolic calcium causes relaxation of vascular smooth muscle and vasodilation.

Local levels of oxygen tension will also affect microcirculatory regulation. Under normal circumstances, capillary blood has a significantly lower PO_2_ (5–12 mm Hg) compared to arteriolar blood (~60 mm Hg). This occurs because of early off-loading in precapillary tissues, as well as endothelial consumption and countercurrent exchange with venous flow (16). An increase in precapillary tissue PO_2_ will promote vasoconstriction, whereas a decrease will result in vasodilation, largely through release of nitric oxide (NO). In addition to this role, NO has an impact on microvascular tone in a number of other circumstances (16). Normally, the constitutive form of nitric oxide synthetase (cNOS) is responsible for maintaining a basal level of NO and modulating vascular tone. Further, an increase in blood viscosity, which occurs in diseases such as polycythemia vera or severe hemoconcentration, results in elevated shear stress on the vascular endothelium. Through mechanotransduction shear stress serves to increase cNOS activity, resulting in increased release of NO, and associated vasodilation (Figure 3). The opposite would occur in severe anemia/hemodilution with significant reduction in red cell mass and blood viscosity (17). The inducible form of nitric oxide synthetase (iNOS) can be produced by the endothelial cells when triggered by the inflammatory cascade. Prostacyclin-induced vasodilation may also play a role in the normal response to hypoxia, especially when NO is blocked there is shear stress (18).

## Microvascular Changes With Trauma and Hemorrhagic Shock

Given the complex nature of the microcirculation and its multifactorial regulation, it stands to reason that microvascular perfusion will be altered in varying ways in response to disease states. In the face of tissue injury, hypotension, and impaired oxygen delivery, it can be expected that there will be activation of both systemic and local compensatory and pathological mechanisms. While some of this response has already been described in a general sense, what follows is more specifically related to microvascular changes secondary to trauma and hemorrhage.

The initial systemic response to tissue trauma, pain, and hemorrhage is largely driven by the sympathetic nervous system leading to release of epinephrine and norepinephrine. The resulting influx of catecholamines will promote vasoconstriction, particularly in the large arterioles (70–150 μm) supplying skeletal muscle (19). Smaller arterioles (10–25 μm) have a more varied response, with constriction to some beds and dilation in others. This next level allows for finer regulation of microcirculatory flow based on metabolic demand and the “essential nature” of the associated organ/tissue bed (e.g., heart and brain).

Local factors caused by trauma and hemorrhage will also cause changes at the level of the small arterioles. Decreased oxygen delivery and tissue hypoxia will tend to cause vasodilation. However, this is offset by inhibition of eNOS (and thereby release of NO) in the early stages of trauma/hemorrhage. With time, however, there is upregulation of iNOS (from both tissue damage and inflammatory mediators) and more tendency for vasodilation (20). In addition, tissue injury and the inflammatory response will promote release of assorted vasoactive mediators with mixed effects. Progressive acidosis and accumulation of cellular metabolites (particularly in the terminal stages of shock) will tend to promote vasodilation and serve to undo systemic vasoconstrictive efforts. Despite the vasodilation, the decrease in driving pressure in the face of hypotension ultimately leads to stagnation of blood flow.

It is also important to consider the potential role of the venous circulation on microvascular perfusion. In the initial response to trauma and hemorrhage, catecholamine-induced venoconstriction, albeit limited, serves to decrease venous capacitance and encourage return of blood to the heart. However, as shock progresses there will be a general relaxation (through similar mechanisms described above), and pooling of blood in venous circulation. This downstream stagnation can then negatively impact blood flow across the capillary bed.

There are several other factors that can contribute to impaired microcirculatory flow. With trauma, inflammation, and shock there can be significant swelling of vascular endothelial cells secondary to increased membrane permeability, acidosis, and impaired electrolyte transport from failure of ATP-dependent channels (21). Given capillary luminal size, further reduction from endothelial swelling can have a significant negative impact on capillary blood flow. Endothelial edema causes decreased release of prostacyclin and NO and increased release of endothelin and thromboxane; the net effect of which is upstream vasoconstriction and a further reduction in capillary flow. Another contributing factor can be decreased red blood cell (RBC) deformability secondary to oxidative injury, ATP depletion, cell membrane injury, and cellular dehydration (21). As RBCs are typically slightly larger than the capillary lumen, folding is necessary for them to effectively move through the microcirculation. Impaired deformability, along with aggregation, can lead to capillary plugging, or shearing injury/destruction of RBCs. Along similar lines, increased leukocyte rigidity, activation, and endothelial adherence can also result in arteriolar and capillary plugging. Lastly, microthrombi formed secondary to tissue/endothelial injury, inflammatory response and hypercoagulability can lodge at various levels of the microcirculation and impede downstream flow.

These upstream and downstream effects will ultimately serve to impact capillary flow and delivery of oxygen and nutrients. Vasoconstriction will lead to shunting of blood away from capillary circulation (decrease vessel number) and hypotension, vasodilation, and obstruction will lead to stagnation (decreased flow). In addition, these abnormalities can persist for an extended period after resuscitation has been achieved, even when macrovascular parameters have been normalized (21, 22).

Shedding of the endothelial glycocalyx has been seen in experimental rodent models of non-traumatic hemorrhagic shock, although the changes are independent of increased vascular endothelial permeability (23–25).

## Microvascular Changes With Sepsis

**Figure 4:**
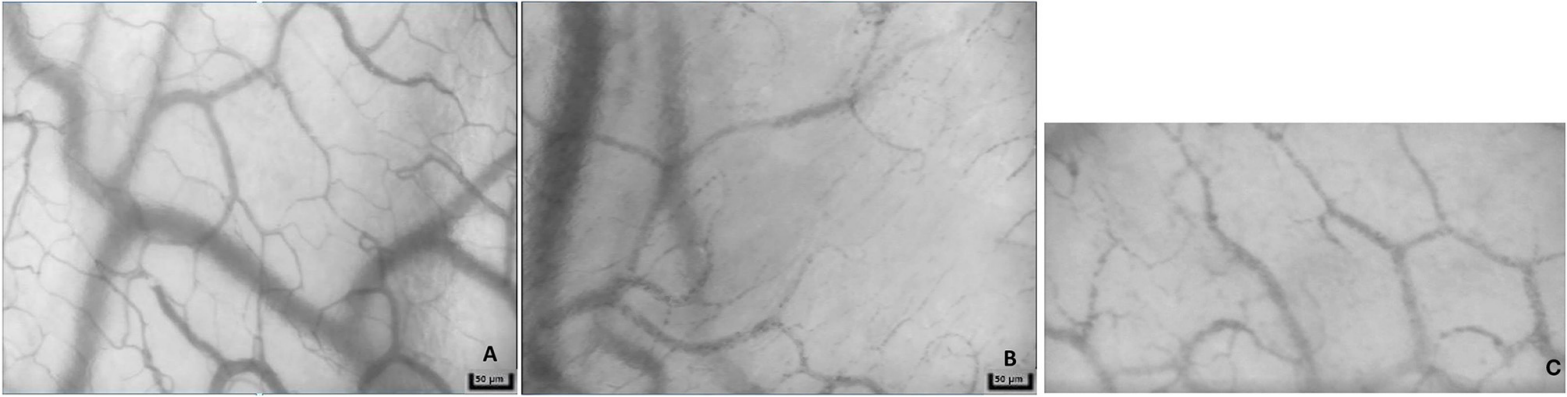
Representative images from a sidestream dark-field microscopy device from a healthy dog **(A)**, dog in hemorrhagic shock **(B)**, and dog in septic shock **(C)**. Note the decreased density of capillaries in patients with shock.

The pathophysiological syndrome of sepsis characterized by the progression of illness severity and potentially culminating in septic shock (26). Septic shock is described in human medicine as the presence of refractory hypotension, hyperlactatemia, and organ dysfunction that persists despite aggressive fluid resuscitation (27). The rapid progression from sepsis to septic shock and organ dysfunction is poorly understood; profound changes in the macro- and microcirculation are believed to contribute to the development of organ failure and subsequent death (28–31). Microcirculatory alterations include a decreased microvascular density and perfusion along with increased capillary flow heterogeneity (Figure 4C and Supplementary Video 3) (28, 29, 32). These changes have been found to precede macrocirculatory changes in septic humans and microcirculatory improvement has also been correlated with improved survival (28, 29, 32, 33). Early microcirculatory changes were a stronger predictor of outcome than any macrocirculatory variable in septic human patients (34).

Various factors likely contribute to the microcirculatory derangements in septic patients, including hypovolemia, endothelial cell dysfunction secondary to adhesion molecule expression, increased white blood cell adhesion, degradation of the endothelial glycocalyx, connexin uncoupling, vascular hyperpermeability, formation and deposition of microthrombi, loss of vasomotor autoregulation and reactivity, changes in local perfusion pressure and flow, and shunting of oxygen to hyperperfused capillary beds (35).

The sublingual microcirculatory derangements appear to correlate with microcirculatory changes in other organs such as the intestines and kidneys in experimental and clinical studies (36–38). Microcirculatory changes in healthy, anesthetized dogs correlate with macrocirculatory measurements of perfusion, although dogs with hemorrhagic shock do not maintain this hemodynamic coherence (39–41). Correlations in septic dogs has not yet been published.

The degraded glycocalyx layer becomes thin and sparse during sepsis, thus enabling plasma proteins and fluid to move across the vascular wall and into the interstitium (42, 43). Degradation of the glycocalyx likely occurs secondary to inflammation and increased circulating “sheddases” such as metalloproteinases, heparinase, and hyaluronidase which are activated by reactive oxygen species and proinflammatory cytokines (44). Specific changes to the endothelial glycocalyx of the lung in septic rodent models include peeling away from the endothelial surface and form spherical bodies that are visualized at the site of damage (45). An decrease in the thickness of the endothelial surface layer was seen in human patients suffering from sepsis and correlated with severity of critical illness. However, there was not an association between this thickness and microcirculatory parameters such as flow index and the proportion of perfused vessels (46).

## Monitoring of the Microcirculation vs. Macrocirculation

There is mounting evidence in human medicine to suggest that goal directed therapy aimed at maximizing macrocirculatory parameters may not improve outcome (47–49). Despite preservation of total blood flow to organs, heterogeneity of microcirculatory flow can lead to hypoxic zones (29, 50). Several techniques have now been developed to assess the microcirculation to investigate its role as a diagnostic, prognostic, and monitoring tool, including laser Doppler, near-infrared spectroscopy, and videomicroscopy (51). As mentioned above, macrocirculatory hypoperfusion is associated with microcirculatory derangements; however, dysfunction of the microcirculation may occur despite normal macrocirculatory indices. This loss of hemodynamic coherence can lead to hyperlactatemia and acidemia despite normal perfusion parameters (also known as cryptic shock) (52). When patients are in shock, normalization of cardiovascular parameters may not equate to improvements in microcirculatory perfusion, as evidenced in both clinical and experimental studies (22, 29, 32, 34, 53). There are four possible mechanisms for this loss of hemodynamic coherence: (1) increased microcirculatory heterogeneity of perfusion (i.e., secondary to inflammatory cytokines), (2) hemodilution from non-oxygen carrying intravenous fluids, thus decreasing hematocrit and oxygen content, (3) microcirculatory vasoconstriction or tamponade from endogenous/exogenous vasopressors and/or increased venous pressure, ad (4) interstitial edema of tissues secondary to endothelial and glycocalyx damage (i.e., secondary to inflammation or excessive fluid therapy) (54). In this regard, direct assessment of microcirculatory flow, such as with videomicroscopy, could be better suited to determine the presence of derangements, as well as response to fluid resuscitation.

Techniques like side stream dark field (SDF) and incident dark field (IDF) microscopy allow direct imaging of the microcirculation with the ability to quantify vessel density as well as assess quality of flow (55). Both of these technologies involve a hand-held device that emits green light (530 nm) which is absorbed by the hemoglobin of erythrocytes. These illuminated red blood cells show up as a dark density flowing through the microcirculation, resulting in a real-time magnified video with resolution to allow detection of true capillaries (Figures 4A–C, Supplementary Videos 1–3). Where they differ is how the light is emitted and received by the device, with reported differences in image quality and magnification (55). Analysis of the resulting videos generates parameters including total vessel density (TVD), proportion of perfused vessels (PPV), perfused vessel density (PVD), and microvascular flow index (MFI) which reflect the quantity and quality of microvascular flow. In order to allow a more consistent approach to microvascular analysis, consensus criteria for the acquisition and analysis of microcirculatory images using have been established (56).

Despite the availability of direct microvascular imaging for two decades and some evidence in veterinary medicine, (39, 40) it has still not become a standard in monitoring response to fluid therapy. Largely this has to do with some of the limitations of these technologies. The camera is expensive and not widely available. Because of the need for light to transmit and reflect back, only mucosal or serosal surfaces can be used for visualization, and areas with keratinized epithelium or significant pigmentation cannot be examined with this technique. In order to generate diagnostic quality videos for analysis, sufficient pressure has to be applied to the tissue, while avoiding excessive compression of the microvasculature (56). While significant steps have been made toward automating the vascular analysis (previously it was largely a manual effort taking over 1 h per video), accuracy of vessel assignment used for automated calculations remains a challenge. This can result in inconsistency and variability of results. Therefore, while microvascular imaging has significant potential to aid in guiding response to fluid therapy, further refinement of automated analysis and increased availability is needed before its use can become more commonplace.

There are two primary methods for assessing the integrity of the endothelial glycocalyx: measurement of shed glycocalyx components in the plasma or serum (i.e., glycosaminoglycans such as hyaluronan and proteoglycan ectodomains such as syndecan-1) and use of imaging techniques that can be done *in vivo* or *ex vivo*. Damage to the ESL and shedding of the glycocalyx has been shown to occur in a multitude of serious illnesses; the degree of shedding is associated with poor outcomes (57). Potential therapeutic strategies to support repair of a damaged ESL, including fluid therapy prescriptions, is the focus of ongoing research.

The use of circulating biomarkers as an indicator of glycocalyx damage has limitations. These primarily include the variable methodology itself, potential for other sources of the markers since they are not unique to the glycocalyx, and upregulation of some markers with diseases such as inflammation. Further details on glycocalyx biomarkers can be found in “Resuscitative Fluid Therapy and the Endothelial Surface Layer” found elsewhere in this “Fluid Therapy in Small Animals” series.

Imaging of the ESL *in vivo, ex vivo*, and *in vitro* has been performed using multiple methods. These include transmission electron microscopy, (58–60) intravital microscopy, (61, 62) microparticle image velocimetry, (63) confocal laser scanning microscopy or atomic force microscopy, (10) two-photon laser scanning microscopy, (64) and videomicroscopy using handheld devices and various imaging technologies (i.e., orthogonal polarization spectroscopy, sidestream dark-field imaging incident dark-field imaging) (56). More recently, *in vivo* indirect assessments of the glycocalyx have been performed in humans and small animals using the sidestream dark-field microscopic technique in conjunction with specialized, proprietary software that measures the width of the vessel lumen available for red blood cell movement as an indirect assessment of glycocalyx thickness (also known as the perfused boundary region or PBR) (65–68). If there is damage to the ESL, RBC are able to penetrate further toward the endothelium and the PBR increases (69).

## Effects of Fluid Resuscitation on the Microcirculation in Trauma and Hemorrhagic Shock

The nature of traumatic injury and hemorrhage presents significant challenges regarding how to optimize fluid therapy and volume replacement, particularly in the animal with active bleeding. In addition to loss of circulating volume, there are also aspects of progressive anemia, protein loss and coagulopathy. While administration of blood products over crystalloid or colloid is generally recommended in human medicine, (70) limited availability in veterinary medicine can result in greater use of crystalloids or consideration of synthetic colloids. Experimental hemorrhagic shock studies in rodent models suggest a benefit of balanced crystalloids, fresh frozen plasma or concentrated albumin over normal saline for restoration of the endothelial glycocalyx (71, 72). Results have not been consistent, however, Further, albumin and fresh frozen plasma appear to be more protective in most studies when compared to synthetic colloids (73). In addition to the type of fluid, volume and rate of administration are also a subject of debate; there is concern that aggressive fluid administration could serve to exacerbate ongoing hemorrhage, worsen coagulopathy, and damage the endothelial glycocalyx (74, 75). Of further consideration are the implications that fluid type and rate of administration might have on the microcirculation. As previously indicated, improving macrovascular parameters does not always correlate to restoration of microvascular perfusion.

Numerous experimental models and preclinical studies have attempted to assess the impact of various fluids on the microcirculation in patients with hemorrhagic shock. A systematic review of many of these studies, totaling 71 articles between 1990 and 2015, evaluated an assortment of fluid comparisons including blood products, hemoglobin-based oxygen carriers, crystalloids and colloids (76). Major findings of this analysis suggested that improved microcirculation was found with solutions containing hemoglobin vs. those without, those that were hyperoncotic vs. those that were not, and those that were hyperviscous vs. those that were not (76). In fitting with this, a recent study in sheep comparing hydroxyethyl starch (HES) and saline found improved hemodynamic coherence with HES, whereas saline only improved macrovascular parameters (77). However, concerns regarding the potential for acute kidney injury with synthetic colloids has served to significantly reduce their use in both human and veterinary medicine. What remains to be determined is whether use of a limited acute volume expansion carries a similar risk. In a human clinical study of patients with traumatic hemorrhagic shock, it was shown that the presence of microvascular derangement at the time of presentation that persisted after resuscitative efforts was more predictive of progression to multiple organ dysfunction syndrome than other more traditional parameters (like lactate and blood pressure) (78). This was true regardless of the type of resuscitative fluid used.

In hemorrhagic shock and trauma, there is potentially even greater interest in specifically looking at the impact of blood transfusion on restoring or maintaining microvascular perfusion. One study in human patients showed that administration of packed red blood cells helped to improve patients with microvascular parameters that were initially decreased, however there was no change or reduction in patients that started with normal values (79). Another pilot study showed improvement in microvascular parameters with red blood cell transfusion of one unit despite no change in macrovascular parameters or hemoglobin levels (80). Interestingly, these changes were negatively correlated with pre-transfusion microvascular parameters, again suggesting that the worse the microvascular impairment, the more significant the improvement. However, duration of red blood cell storage could alter the impact of red cell transfusion on microvascular perfusion. It has been demonstrated that aged red cell units may have increased levels of free hemoglobin which can serve to scavenge NO and worsen microvascular blood flow (81). Future studies evaluating the use of plasma for resuscitation from trauma are underway; initial data suggests that plasma therapy may play a beneficial role in ameliorating immunomodulatory dysfunction and trauma-induced endotheliopathy (82).

## Effects of Fluid Resuscitation on the Microcirculation in Sepsis

Intravenous fluid therapy has been one of the cornerstones of treatment for sepsis spanning many decades. Although fluid resuscitation may improve microvascular perfusion, this effect is not predictable and likely depends in part on the timing of administration; microvascular perfusion is improved if fluids are given within 12–24 h of diagnosing sepsis but may be less effective or even deleterious when given at later stages according to one study (83). However, additional studies found that bolus fluid therapy worsened survival in people with sepsis (84, 85). Humans with abdominal sepsis displayed an increase in mean arterial blood pressure, cardiac index, and sublingual microcirculatory red blood cell velocity following a fluid challenge; however, the intestinal microcirculatory indices did not change (86). The role of fluids in the augmentation of septic endothelial dysfunction and glycocalyx damage is the focus of current investigation (87). Adverse outcomes have also been linked to fluid therapy and fluid balance, increasing the need to determine the pathogenesis of these findings (88–93). There is evidence to suggest that intravenous crystalloid and colloid fluid administration promotes endothelial glycocalyx degradation in endotoxemic sheep (94) and humans with sepsis (74, 87). Although the exact mechanism is still unknown, there are several possible etiologies: (1) acute vascular stretching along with inflammatory mediators could stimulate endothelial expression of glycocalyx-shedding matrix metalloproteinase, (95) (2) oscillatory shear stress-induced increases in cathepsin L activation (an enzyme that may be involved in post-translational activation of endothelial heparinase), (96) (3) direct activation of circulating leukocytes and trigger neutrophil-elastase glycocalyx destruction, (97–99) and (4) atrial natriuretic peptide which has been found to induce glycocalyx damage in some human studies (26, 100, 101).

The effect of the type of fluid administered may also play a role in causing glycocalyx damage with sepsis. Both clinical and preclinical data suggest that balanced crystalloids, albumin, fresh frozen plasma, and synthetic colloids may be less injurious compared to isotonic saline (102–104). It is possible that albumin preserves the glycocalyx and may be more beneficial compared to isotonic crystalloids in experimental studies, (105, 106) but results of glycocalyx evaluation following albumin therapy in septic patients are not yet available. Albumin therapy in the form of concentrated albumin or plasma products has sparked interest for its potential ability to protect the glycocalyx due to its ability to carry erythrocyte-derived sphingosine-1-phosphate to the endothelium; this may mediate glycocalyx recovery by suppressing metalloproteinase activity (107–109). It has even been suggested that individual patients should receive tailored fluid treatment plans based on admission markers of endothelial glycocalyx damage to avoid the deleterious consequences of fluid administration to patients with high risk for vascular leakage and subsequent organ dysfunction (109).

## Conclusion

In conclusion, the hemodynamic coherence between the macro- and microcirculation is often poor in shock states such as hemorrhage or sepsis. Various diseases lead to vascular changes that may not be readily apparent with current monitoring strategies. Therefore, intravenous fluid resuscitation strategies must take into account not only microcirculatory parameters such as systemic arterial blood pressure, but also downstream measures and/or microcirculatory assessments of the patient's response to treatment. Continued research focusing on the effects or different fluid therapy prescriptions on the macro- and microcirculation/endothelial surface layer in various disease states, the goals and timing of its administration, and ultimately outcome of the patients will likely change fluid therapy in the future.

## Author Contributions

Both authors listed have made a substantial, direct and intellectual contribution to the work, and approved it for publication.

## Conflict of Interest

The authors declare that the research was conducted in the absence of any commercial or financial relationships that could be construed as a potential conflict of interest.

## Supplementary Material

Supplementary Video 1 Sidestream dark-field microscopy from a healthy dog.

Supplementary Video 2 Sidestream dark-field microscopy from a dog with hemorrhagic shock.

Supplementary Video 3 Sidestream dark-field microscopy from a dog with septic peritonitis.
